# Phosphofructo-2-kinase/Fructose-2,6-bisphosphatase Modulates Oscillations of Pancreatic Islet Metabolism

**DOI:** 10.1371/journal.pone.0034036

**Published:** 2012-04-20

**Authors:** Matthew J. Merrins, Richard Bertram, Arthur Sherman, Leslie S. Satin

**Affiliations:** 1 Department of Pharmacology and Brehm Diabetes Center, University of Michigan, Ann Arbor, Michigan, United States of America; 2 Department of Mathematics and Programs in Neuroscience and Molecular Biophysics, The Florida State University, Tallahassee, Florida, United States of America; 3 Laboratory of Biological Modeling, The National Institute of Diabetes and Digestive and Kidney Diseases, National Institutes of Health, Bethesda, Maryland, United States of America; Boston University, United States of America

## Abstract

Pulses of insulin from pancreatic beta-cells help maintain blood glucose in a narrow range, although the source of these pulses is unclear. It has been proposed that a positive feedback circuit exists within the glycolytic pathway, the autocatalytic activation of phosphofructokinase-1 (PFK1), which endows pancreatic beta-cells with the ability to generate oscillations in metabolism. Flux through PFK1 is controlled by the bifunctional enzyme PFK2/FBPase2 (6-phosphofructo-2-kinase/fructose-2,6-bisphosphatase) in two ways: via (1) production/degradation of fructose-2,6-bisphosphate (Fru2,6-BP), a potent allosteric activator of PFK1, as well as (2) direct activation of glucokinase due to a protein-protein interaction. In this study, we used a combination of live-cell imaging and mathematical modeling to examine the effects of inducibly-expressed PFK2/FBPase2 mutants on glucose-induced Ca^2+^ pulsatility in mouse islets. Irrespective of the ability to bind glucokinase, mutants of PFK2/FBPase2 that increased the kinase:phosphatase ratio reduced the period and amplitude of Ca^2+^ oscillations. Mutants which reduced the kinase:phosphatase ratio had the opposite effect. These results indicate that the main effect of the bifunctional enzyme on islet pulsatility is due to Fru2,6-BP alteration of the threshold for autocatalytic activation of PFK1 by Fru1,6-BP. Using computational models based on PFK1-generated islet oscillations, we then illustrated how moderate elevation of Fru-2,6-BP can increase the frequency of glycolytic oscillations while reducing their amplitude, with sufficiently high activation resulting in termination of slow oscillations. The concordance we observed between PFK2/FBPase2-induced modulation of islet oscillations and the models of PFK1-driven oscillations furthermore suggests that metabolic oscillations, like those found in yeast and skeletal muscle, are shaped early in glycolysis.

## Introduction

The plasma insulin pulses arising from pancreatic beta-cells [1] are strongest in the portal blood system, more effective than continuous administration in suppressing hepatic glucose output [2], [3], and lost in diabetics and their near relatives [4], [5]. Befitting their role as metabolic sensors for the organism, beta-cells adjust their metabolic output in accordance with plasma glucose concentration, and not just their own energy requirements. Glucose sensing is mediated by glucokinase, which is rate-limiting and controls metabolic flux into phosphofructokinase-1 (PFK1) [6]. As the archetypal metabolic pathway, the initial steps of glycolysis have been biochemically characterized and computationally modeled, leading to the proposal that PFK1 activity determines the oscillatory activity of beta-cells [7], [8] and governs the downstream oscillations observed in metabolism (mitochondrial NADH and O_2_, ATP [9]–[11], electrical activity (K_ATP_ and Ca^2+^ channel activity) [12], and finally, pulsatile insulin release.

The bifunctional enzyme PFK2/FBPase2 (phosphofructo-2-kinase/fructose-2,6-bisphosphatase) is uniquely positioned to alter flux through PFK1 in two complementary ways. First, PFK2/FBPase2 is the sole catalyst for the production and degradation of fructose 2,6-bisphosphate (Fru2,6-BP), which allosterically activates PFK1 even more potently than its own product, fructose 1,6-bisphosphate (Fru1,6-BP), overriding the feedback inhibition of PFK1 by ATP and citrate [13]–[16]. Second, PFK2/FBPase2 has been proposed to bind and directly activate glucokinase (GK) [17]–[20], which controls the flux of fructose 6-phosphate (Fru-6P) substrate into PFK1 [6]. In support of this latter pathway, insulin-secreting cells overexpressing GK and PFK2/FBPase2 showed enhanced glucokinase activity, glycolytic flux, and glucose-stimulated insulin secretion [21], [22], while downregulation of PFK2/FBPase2 using siRNAs in INS-1E cells decreased glucokinase activity and insulin secretion [23]. While providing a biochemical framework, these studies have not addressed pulsatility.

The goal of this study was to determine the effects of the positive feedback provided by PFK2/FBPase2 and its product Fru2,6-BP on islet oscillations. Do they amplify or attenuate oscillations? What effect do they have on the oscillation frequency? Which of the two feedback pathways dominates? Using data obtained from time-lapse imaging of islet Ca^2+^ oscillations under conditions of altered PFK2/FBPase2 activity, we show that PFK2/FBPase2 modulates the frequency of islet oscillations, primarily through the effect of its product Fru2,6-BP on PFK1. These data can be understood in terms of the influence of Fru2,6-BP on endogenous glycolytic oscillations, as we demonstrate with a computational model.

## Results

### Islet Ca^2+^ oscillations are shaped by mutants of PFK2/FBPase2

**Figure 1:**
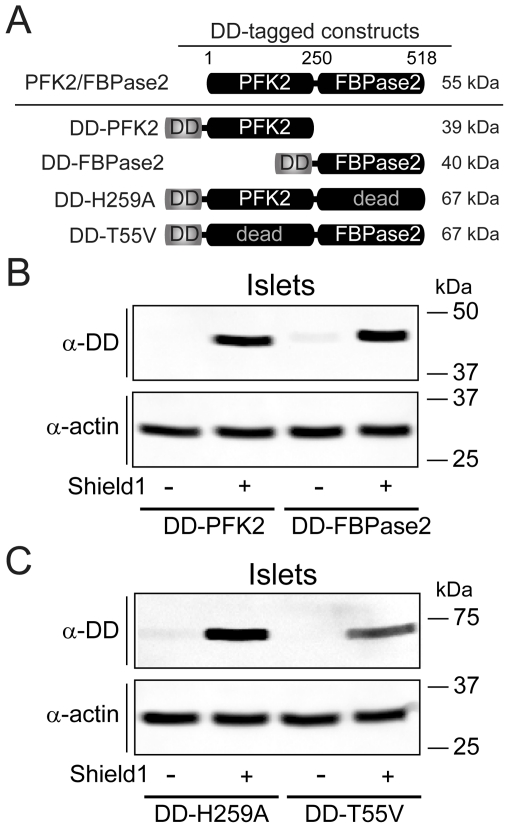
Inducible expression of degradation-domain (DD)-tagged mutants of PFK2/FBPase2 in pancreatic islets. (A) Schematic of DD-tagged mutants of PFK2/FBPase2. (B and C) Western blots of islets transduced with DD-PFK2 and DD-FBPase2 truncations mutants (*B*), or DD-tagged phosphatase-dead PFK2/FBPase2 (DD-H259A) and kinase-dead PFK2/FBPase2 (DD-T55V) point mutants (*C*), treated with either vehicle (0.1% DMSO) or 1 µM Shield1, and visualized with an anti-DD antibody. Representative of three independent experiments (50 islets per treatment).

To test the effects of PFK2/FBPase2 on islet oscillations we carried out a series of experiments in mouse pancreatic islets expressing adenovirally-delivered mutants of the islet isoform of PFK2/FBPase2, a *pfkfb2* gene product [23], [24]. Each of the four PFK2/FBPase2 mutants used in this study (Fig. 1A) was N-terminally tagged with a Degradation Domain (DD), which permits transcription and translation but prevents the accumulation of functional protein in the cytosol due to rapid proteosomal degradation; degradation is inhibited in a controlled way by the addition of a small cell-permeant molecule, Shield1 [25]. This inducible expression system allowed direct comparison between virally-transduced islets from the same isolation treated with either 1 µM Shield1 or vehicle (0.1% DMSO), neither of which had effects when applied alone.

Our first two manipulations focused on the alteration of Fru2,6-BP levels using truncation mutants of PFK2/FBPase2 in which either the C-terminal phosphatase or the N-terminal kinase domain was selectively deleted (DD-PFK2 and DD-FBPase2, respectively) [26]–[30]. As a complementary approach, we constructed DD-tagged phosphatase-dead (H259A) and kinase-dead (T55V) mutants of PFK2/FBPase2; these point mutants target the active site of each domain while leaving the opposing reaction site catalytically active [31]–[33]. Thus, the DD-H259A mutant increases [34] and the DD-T55V decreases [35] the kinase/bisphosphatase ratio. In addition to altering Fru2,6-BP levels, the H259A and T55V mutants have been implicated in the direct binding and activation of GK [17], [19]–[21]. However, because full-length PFK2/FBPase2 is required for the interaction with GK (see the co-immunoprecipitation and FRET experiments in Figure S1), a comparison between the point mutants and truncation mutants provides a method for discerning possible effects of PFK2/FBPase2 on PFK1 from effects on GK.

From the Western blots shown in Figures 1B and 1C it can be seen that the DD-tagged constructs were largely degraded in the absence of Shield1, whereas the presence of 1 µM Shield1 was permissive for an increase in protein levels. We found the kinetics of the increase in DD-tagged proteins upon Shield1 treatment to be slow. Based on imaging of Min6 cells expressing DD-mCerulean (not shown), increases in expression with Shield1 were not observable in < 1 hr unless undesirably high viral titers were used. In islets, 3D confocal imaging of the PFK2/FBPase2 mutants expressing an IRES2-linked mCherry indicated penetration of the adenovirus up to 17 µm (see the video file in Movie S1 online), about two cell layers deep. This would be expected to cover ∼70% of a 100 µm islet, but this is likely an overestimate due to incomplete transduction. Thus, the success of the experiments relies on intra-islet synchronization due to (1) gap junction-mediated electrical coupling between a beta-cell and its 5–6 adjacent neighbors [36], as well as (2) bidirectional coupling between membrane Ca^2+^ flux and metabolism [37], [38].

**Figure 2:**
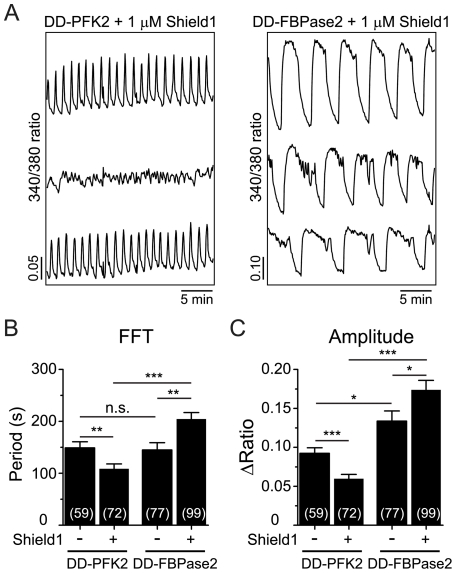
Effect of expressing PFK2/FBPase2 truncation mutants on islet Ca^2+^ oscillations. (A) Representative recordings of islet Ca^2+^ in the presence of the indicated constructs and 1 µM Shield1; note the difference in scaling. (B and C) Average oscillatory period (*B*) and amplitude (*C*) as determined from >20 min of intracellular Ca^2+^ measurements of transduced islets treated with vehicle or Shield1. Numbers in parentheses indicate the number of islets measured in ≥4 independent experiments. 11.1 mM glucose was used in all experiments. * *p*<0.05, ** *p*<0.01, *** *p*<0.001.

Time-lapse imaging was used to compare the Ca^2+^ oscillations induced by 11.1 mM glucose in islets that had been transduced with either DD-PFK2 or DD-FBPase2. While transduction or vehicle alone did not affect the islet oscillations (not shown), from the representative recordings of Shield1-treated islets shown in Figure 2A, it can be seen that islets expressing DD-PFK2 exhibited Ca^2+^ oscillations that were faster and reduced in amplitude compared to those expressing DD-FBPase2. Furthermore we observed a synchronous response from the entire islet, indicating crosstalk between the transduced and untransduced cells. Compared with vehicle treatment, the application of Shield1 dramatically reduced the mean oscillatory period and amplitude of islet Ca^2+^ oscillations by 27% and 35%, respectively, in DD-PFK2 expressing islets (Figs. 2B, C). In contrast, the average period and amplitude of DD-FBPase2 transduced islets were increased by 41% and 30%, respectively, upon Shield1 treatment, and exhibited a ∼2-fold increased period and ∼3-fold increased amplitude relative to oscillations in DD-PFK2 expressing islets (Figs. 2B, 2C). Taken together, these experiments indicate that the oscillatory behavior of islets is modulated by Fru2,6-BP levels.

**Figure 3:**
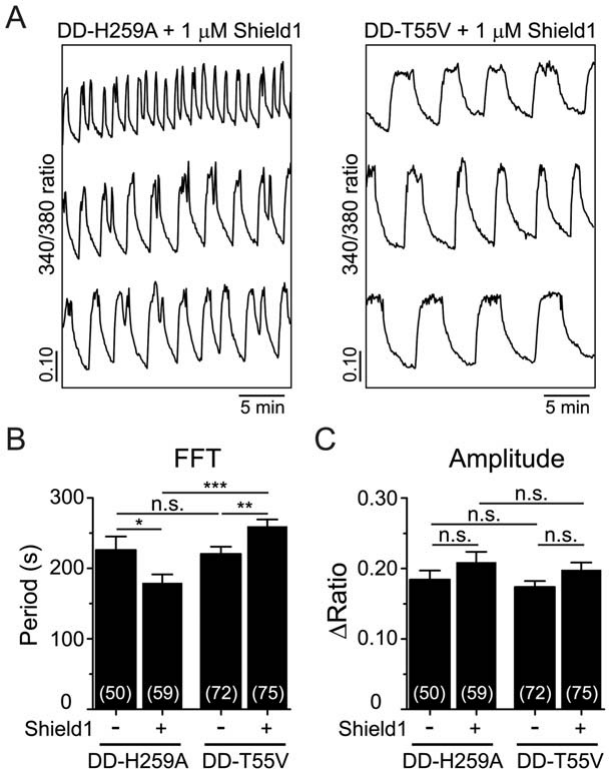
Effect of expressing PFK2/FBPase2 point mutants on islet Ca^2+^ oscillations. (A) Representative recordings of islets Ca^2+^ in the presence of the indicated constructs and 1 µM Shield1. (B and C) Average oscillatory period (*B*) and amplitude (*C*) were determined from >20 min of intracellular Ca^2+^ measurements of transduced islets treated with vehicle or Shield1. Numbers in parentheses indicate the number of islets measured in ≥4 independent experiments. 11.1 mM glucose was used in all experiments. * *p*<0.05, ** *p*<0.01, *** *p*<0.001.

We then compared the Ca^2+^ oscillations induced by 11.1 mM glucose in islets that had been transduced with either DD-H259A or DD-T55V. As exemplified by the traces shown in Fig. 3A, Shield1-treated islets expressing phosphatase-dead DD-H259A exhibited [Ca^2+^]_i_ oscillations that were significantly faster (by 44%, on average) than those expressing kinase-dead DD-T55V. Compared with control, Shield1 treatment induced a 26% decrease in the average period of the Ca^2+^ oscillations of DD-H259A transduced islets, whereas the DD-T55V transduced islets exhibited a comparable increase in period following Shield1 treatment (18%) (Fig. 3B). The amplitude of the oscillations was not affected in either case (Fig. 3C). It is notable that even in the absence of Shield1 the initial periods of the oscillations vary (*cf.*, Figs. 2B and 3B). This variability is expected and most likely due to ‘islet imprinting’. That is, the average period of slow oscillations tends to be consistent between islets from the same mouse while varying considerably (from less than 1 min to about 7 min) between mice [39], [40]. Consequently, as the controls for Figs. 2 and 3 were done on different days with different mice, one would expect significant variation in frequencies. Nonetheless, the GK-interacting mutants (H259A and T55V), which increase and decrease Fru2,6-BP levels, respectively, had opposite effects on oscillation period whereas they would have been predicted to have the same effect on GK, indicating that the predominant effect of PFK2/FBPase2 on islet oscillatory behavior appears to occur through its actions on PFK1, rather than GK.

### Fructose-2,6-bisphosphate can speed up or terminate glycolytic oscillations: model simulations

We have shown that expression of PFK2/FBPase2 mutants known to elevate the levels of Fru2,6-BP above physiologic concentrations resulted in higher-frequency oscillations, while reducing Fru2,6-BP had the opposite effect. In this section, we use a mathematical model of PFK1-mediated glycolytic oscillations [41] to illustrate how this might occur, and further, we show that levels of Fru2,6-BP that are insufficient to terminate the oscillations outright may be able to increase their frequency while decreasing their amplitude, akin to the experimental results shown in Figs. 2 and 3. We then examine the consequences of a simulated increase in Fru2,6-BP on a more complete model of the pancreatic β-cell.

**Figure 4:**
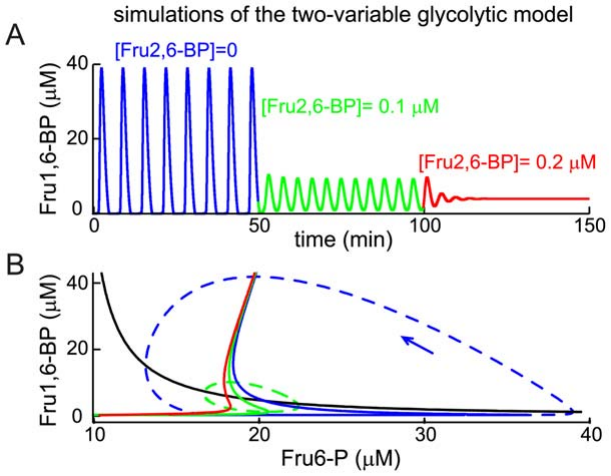
Fru1,6-BP oscillations produced by the two-variable glycolytic oscillator model are modified by Fru2,6-BP. (A) Addition of Fru2,6-BP can terminate the oscillations (red), or at an intermediate level (green), make the oscillations faster and smaller. (B) The Fru6-P nullcline (black) is unaffected by Fru2,6-BP, but Fru2,6-BP pulls together the knees of the Fru1,6-BP nullcline (shown as solid blue, then green and then red lines as Fru2,6-BP is increased as in (A)), eventually stabilizing the equilibrium that exists at the intersection of the Fru6-P and Fru1,6-BP nullclines. The orbit of the oscillation with Fru2,6-BP = 0 is shown (blue dashed line) along with an arrow indicating the orientation of the orbit.

The model we used to simulate beta-cell glycolytic oscillations was the same used in our previous studies [37], [38]. It consists of differential equations for the concentrations of the PFK1 substrate Fru6-P and the product Fru1,6-BP:
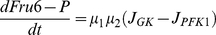
(3)

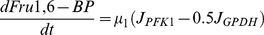
(4)where 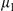 is a scaling factor that adjusts oscillation frequency in this stand-alone glycolytic model for the removal of the calcium feedback that exists in the full Dual Oscillator Model (DOM) used in the next section (see Methods; we use 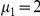 to produce ∼7 min oscillations in the absence of Fru2,6-BP) and 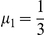 relates Fru6-P and glucose 6-phosphate concentrations. The enzymatic fluxes are *J_GK_* for GK (a constant input to the system), *J_GPDH_* for glyceraldehyde 3-phosphate dehydrogenase, and *J_PFK1_* for PFK1, which converts Fru6-P to Fru1,6-BP. The latter reaction is allosterically stimulated by Fru1,6-BP and inhibited by ATP (which we hold constant at 800 µM in Fig. 4A). It is the positive feedback on PFK1 provided by Fru1,6-BP, combined with a slower reduction in the substrate Fru6-P, that endows the system with the ability to oscillate.

Figure 4A (blue) shows the oscillation in Fru1,6-BP produced by the model when 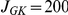 μM ms^-1^ and [Fru2,6-BP] = 0. The basis for the oscillation can be understood in terms of the nullclines of the two variables. These are the curves shown in the Fru6-P/Fru1,6-BP phase plane (Fig. 4B) where the derivatives of the variables are zero. The Fru6-P nullcline 
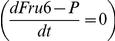
 is the black curve, which shows that Fru6-P declines when Fru1,6-BP rises because the former is converted into the latter. The Fru1,6-BP nullcline 
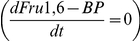
 is the solid blue curve, which is S-shaped, reflecting the strong nonlinear effect of Fru1,6-BP on PFK1. Focusing on the bottom leg of the S, we see that the steady state response of Fru1,6-BP to a rise in Fru6-P is negligible until Fru6-P crosses the threshold represented by the sharp lower knee of the S, which results in a rapid rise in Fru1,6-BP as it is produced by PFK1 and a fall in Fru6-P as it is consumed. This threshold crossing is responsible for the surge of Fru1,6-BP in the blue portion of the time course in Fig. 4A. The Fru1,6-BP nullcline finally turns subtly back to the right, creating an upper knee as very high Fru6-P drives a maintained increase in Fru1,6-P.

These curves divide up the Fru6-P/Fru1,6-BP plane into sectors in which the derivatives have a fixed sign and general direction of flow. Equilibrium occurs at the intersection of the nullclines where both derivatives are 0, and depending on its location, the equilibrium may be stable or unstable. When the equilibrium is on the middle branch of the S-shaped curve, the equilibrium may be unstable (intersections on the lower or upper branches produce stable equilibria). In fact, this is the case, and the instability of the equilibrium gives rise to a stable oscillatory solution (indicated by the blue dashed line in Fig. 4B).

Fructose-2,6-bisphosphate enters the PFK1 reaction much as Fru1,6-BP does, but it binds with a higher affinity (the rate *J_PFK1_* is increased by Fru2,6-BP, which competes with Fru1,6-BP; see Methods for model implementation). When Fru2,6-BP is increased from 0 to 0.1 µM the glycolytic oscillation amplitude is greatly reduced while its frequency is increased (Fig. 4A, green). This is due largely to a change in the shape of the Fru1,6-BP nullcline (Fig. 4B, green solid curve); the lower knee of the curve is shifted leftward, so that the horizontal distance between upper and lower knees is now greatly reduced. In other words, Fru2,6-BP lowers the threshold for activation of PFK1 by Fru1,6-BP. Consequently, Fru6-P does not have to build up as much to trigger a surge of Fru1,6-BP, which both shortens the time to the next pulse and reduces the size of the oscillation orbit, which encloses the two knees (Fig. 4B, green dashed curve). Thus, the result is a smaller orbit, a smaller oscillation amplitude, and a faster oscillation. If the concentration of Fru2,6-BP is increased further, to 0.2 μM, the lower knee moves to the left of the upper knee, so that the Fru1,6-BP nullcline is no longer S-shaped (Fig. 4B, red). When this occurs, the equilibrium becomes stable and the oscillatory solution disappears (Fig. 4A, red). In this case, there is no threshold and Fru-6P is continually converted to Fru1,6-BP. The high rate of activity of PFK1 draws off Fru-6P as fast as it is produced, so that there is constant production of Fru1,6-BP rather than cycles of Fru6-P depletion followed by recovery of Fru6-P and a surge of Fru1,6-BP production.

In summary, Fru2,6-BP speeds up and eventually terminates glycolytic oscillations by taking the curvature out of the Fru1,6-BP nullcline. Fru2,6-BP stimulates PFK1, making the PFK1-mediated oscillation faster. However, if PFK1 is stimulated too much an oscillation cannot be sustained, and instead the system comes to rest with an elevated level of Fru1,6-BP.

We next use the Dual Oscillator Model (DOM) [8], [38] of the pancreatic β-cell to predict the effect of Fru2,6-BP on islets, which utilize the muscle type (M-type) of PFK1 as well as other isoforms [42]. This model consists of an electrical oscillator coupled to the slower glycolytic oscillator illustrated in Fig. 4 and can produce a range of behaviors, including compound bursting (fast Ca^2+^ oscillations superimposed on slow oscillations) and slow bursting (Ca^2+^ oscillations with period of 3–7 min). We examine the effect of Fru2,6-BP on slow bursting by incorporating Fru2,6-BP into the PFK1 reaction function, as was done above for the stand-alone glycolytic oscillator model. Unlike above, the Fru2,6-BP level is determined by the PFK2 and FBPase2 reactions. That is,
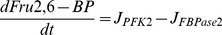
(5)where the reaction terms are given in Methods. In the model, we consider three cases: (1) absence of the PFK2/FBPase2 molecule, so that Fru2,6-BP = 0, (2) PFK2/FBPase2 is present and the ratio of the maximum reaction velocities 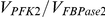 is 0.5, and (3) the latter velocity ratio is increased to 2, increasing the Fru2,6-BP concentration.

**Figure 5:**
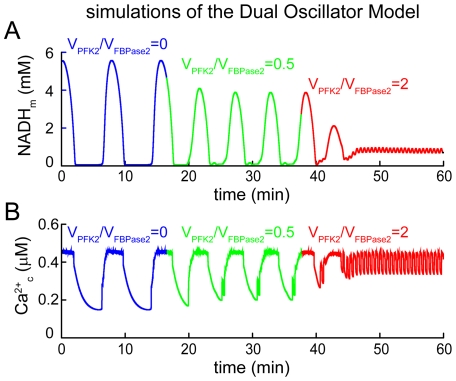
Simulations with the full Dual Oscillator Model. (A) Mitochondrial NADH oscillations occur due to oscillations in glycolysis. These are terminated when the ratio *V_PFK2_/V_PFK2_*, and thus the Fru2,6-BP level, is sufficiently large. (B) Slow Ca^2+^ oscillations are made faster and smaller by the addition of PFK2/FBPase2, with *V_PFK2_/V_PFK2_* = 0.5. They are replaced by fast Ca^2+^ oscillations when *V_PFK2_/V_PFK2_* = 2.

In the absence of PFK2/FBPase2, the DOM produces slow mitochondrial NADH (NADH_m_) oscillations driven by glycolytic oscillations, with a period of approximately 5 min (Fig. 5A). These metabolic oscillations are reflected in oscillations in cytosolic Ca^2+^ (Fig. 5B). When PFK2/FBPase2 is present at low to moderate amounts, the metabolic oscillations become smaller and faster, leading in turn to smaller and faster Ca^2+^ oscillations. However, when 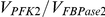 is increased from 0.5 to 2.0, such that more Fru2,6-BP is produced, the metabolic oscillations are terminated (Fig. 5A). This leaves the electrical oscillator intact, so that fast bursting and concomitant fast [Ca^2+^]_i_ oscillations are observed (Fig. 5B). A similar series of behaviors can be produced by the DOM in its compound bursting mode, although in this case there is a smaller reduction in [Ca^2+^] amplitude when 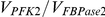.

## Discussion

We have studied the bifunctional enzyme PFK2/FBPase2 in pancreatic islets in order to determine its possible effects on oscillatory activity. PFK2/FBPase2 was manipulated in two different ways: (1) by overexpression of the kinase or phosphatase moieties individually, and (2) by introducing point mutants to selectively eliminate the kinase or phosphatase activities. Since the point mutants are GK-interacting [21], [23] but the truncation mutants are not (Fig. S1), we were able to differentiate the effects of PFK2/FBPase2 acting through Fru2,6-BP from those affecting GK, both which bear on PFK1. The results of the PFK2 and FBPase2 overexpression experiments in Fig. 2 were in good qualitative agreement with the predictions of the Dual Oscillator Model, namely, that enhancing the kinase function made slow oscillations in [Ca^2+^]_i_, a surrogate for metabolic oscillations [11], [37], faster and smaller in amplitude, whereas enhancing the phosphatase function made them slower and larger. The point mutants (Fig. 3) were also consistent with the predictions for oscillation period, but no consistent effect on amplitude was observed. By comparison, it has been shown experimentally that the addition of Fru2,6-BP to rat skeletal muscle extracts exhibiting glycolytic oscillations resulted in the termination of the oscillations if given at a sufficiently high concentration [43]. Those oscillations which were not terminated outright became faster and smaller as Fru2,6-BP was increased. As we measured [Ca^2+^]_i_, we could not determine whether glycolytic oscillations were terminated, but based on the increased frequency seen in islets in which PFK2 was over-expressed, we suspect that this occurred in some cases. Thus, the situation in muscle cells parallels our findings in islets.

### Implications for computational models of the beta-cell

In muscle extracts, glycolytic oscillations are produced by the autocatalytic feedback of Fru1,6-BP onto PFK1 [44]–[46]. The mechanism of these oscillations, which has been well-defined in yeast [44], is activation of PFK1 by its product and the subsequent depletion of its substrate [41], [42], [45], [46]. The period of these oscillations (5–10 min) in muscle resembles the period of slow insulin oscillations [7]. We observed good agreement between the Dual Oscillator Model, which invokes this same mechanism to generate glycolytic oscillations, and the data obtained from time-lapse imaging of islet Ca^2+^ oscillations under conditions of altered PFK2/FBPase2 activity.

The evidence for the PFK1 hypothesis, first proposed by Corkey and Tornheim and colleagues in 1988 (reviewed in [7]) is largely indirect. Thus, the data we report here lend new support to the Dual Oscillator Model. Although we cannot definitively rule out alternative models in which metabolic oscillations occur downstream of glycolysis, such as in the mitochondria [47], or secondary to Ca^2+^ oscillations [48], the specific concordance between the predictions and the measurements is difficult to explain with these other models. For example, if the effect of enhancing PFK2 activity were simply to increase glycolytic flux to the mitochondria, we would not expect an increase in frequency and a reduction in amplitude. Rather, the effect of PFK2 in the experiments was consistent with its role in the model for its product Fru2,6-BP to modulate the threshold for oscillations driven by PFK1.

### Feedback of PFK2/FBPase2 on glycolysis

We confirmed by FRET and immunoprecipitation that full-length PFK2/FBPase2 interacts with GK, albeit weakly (Figure S1). Indeed, both islet and liver isoforms of PFK2/FBPase2 are reported to increase the V_max_ of GK in cell lines [21], [22], which has been proposed to keep substrate available as Fru2,6-BP lowers the PFK1 threshold and increases its activity. However, this interaction is trumped by a much stronger effect of the bifunctional enzyme on PFK1. That is, GK-interacting point mutants had opposite effects on the oscillation period whereas they would have the same effect on GK. A dominant effect of Fru2,6-BP in islets is therefore concordant with the case in hepatocytes [23] since in these cells the same kinase-dead and phosphatase-dead mutants have strongly opposing affects on both Fru2,6-BP levels and glycolytic flux.

We speculate that PFK2/FBPase2 is most helpful to the glucose-sensing capacity of GK in a fasted organism, or in the transition to threshold as glucose is elevated during feeding. In this regard, the liver isoform of PFK2/FBPase2 exhibits a switch-like behavior, with inactivation of the kinase and activation of the bisphosphatase via phosphorylation at Ser-32 in the fasted state [49], which is acutely reversed on refeeding, activating glycolysis both by increasing Fru2,6-BP production and by enhancing glucose phosphorylation [19]. This regulatory site is absent in the beta-cell isoform, which is instead regulated by inactivation of the bisphosphatase at Ser-466 by AMP-activated protein kinase (AMPK) in the low energy state [23]. Nonetheless, PFK2/FBPase2 activity may lower the threshold for beta-cell oscillations – indeed it can left-shift the dose-response curve for insulin release [20] – whereas the oscillatory regime studied in our experiments represents a fed state for the organism, when AMPK activity is low. We leave this possibility for future work.

We conclude that the results reported here are supportive of the main assumptions of the DOM and that PFK2/FBPase2 exerts its effects on metabolic oscillations in beta-cells primarily through the effect of its product Fru2,6-BP on PFK1 rather than through an interaction with GK. It is of interest that whereas the product of PFK2/FBPase2, Fru2,6-BP, affects PFK1 much more strongly than Fru1,6-BP, the product of PFK1, Fru2,6-BP plays second fiddle to Fru1,6-BP. This is because Fru1,6-BP endows PFK1 with threshold properties via auto-catalysis while Fru2,6-BP is not auto-catalytic for PFK1 and only modifies the threshold.

## Materials and Methods

### Culture and adenoviral infection of mouse pancreatic islets

Experiments were carried out using male Swiss-Webster mice (25–30 g) from Charles River Laboratories (Wilmington, MA). Mice were sacrificed by cervical dislocation according to the regulations of the University of Michigan Committee on the Use and Care of Animals (UCUCA), who approved this study under protocol number 10147. Islets were isolated from the pancreas as in [50], and cultured overnight in RPMI1640 supplemented with 10% (v/v) fetal bovine serum (FBS), 100 units/ml penicillin and 100 µg/ml streptomycin (Invitrogen). Groups of 25 freshly-isolated islets were immediately infected with 2000 MOI of each adenoviral construct for 1.5 h in a 95/5% air/CO_2_ incubator at 37°C, followed by removal to fresh culture media lacking virus, but containing either 1 µM Shield1 or vehicle (0.1% DMSO). To estimate infection efficiency, Cherry-labeled cells were compared with islet nuclei stained with Quant-iT PicoGreen (Invitrogen) for 1 h at 37°C and imaged live on a Nikon Instruments A1 Confocal Microscope (Melville, NY).

### Chemicals and expression constructs for live cell imaging

Mouse anti-human FKBP12 monoclonal antibodies, used at 1∶500, were from BD Transduction Labs (cat. 610808), and donkey anti-mouse HRP, used at 1∶5000, was from Jackson Labs (cat. 715-035-150). hield1 was purchased from Cheminpharma (New Haven, CT). The cDNAS for mouse islet/brain PFK2/FBPase2 (in pCMV-Sport6) and human beta-cell GK (in pOT-B7) were obtained from Invitrogen (Carlsbad, CA), and subcloned into a modified entry vector (pENTR1A-DS, Invitrogen) lacking the CmR and ccdB cassettes (pEN), using the In-Fusion Advantage PCR Cloning Kit (Clontech, Mountain View, CA). LR ClonaseII was then used to transfer PFKFB2 and GK upstream or downstream of monomeric cerulean or citrine contained in custom built destination vectors modified from pcDNA6.2-V5/DEST (Gateway System, Invitrogen). The resulting expression vectors (pG-CMV-Cerulean-GK, pG-CMV-GK-Cerulean, pG-CMV-Citrine-PFKFB2, pG-CMV-Cerulean-PFKFB2, pG-CMV-PFKFB2-Citrine, and pG-CMV-PFKFB2-Cerulean), were then used for FRET analysis as donor/acceptor pairs. To facilitate inducible expression of PFKFB2 mutants, the pPTunerN-IRES2-AcGFP vector (ProteoTuner System, Clontech) was purchased from Clontech, and AcGFP was replaced with Cherry from pLoxP-Cherry [51]. The entire expression cassette was then transferred to pEN using the In-Fusion reaction, yielding pEN-PTunerN-IRES2-Cherry. The 12 kDa TunerN cassette, which is referred to above as the degradation domain (DD), is a mutant FKBP12 which facilitates the rapid proteosomal degradation of translated DD-tagged proteins; application of a cell-permeant compound, Shield1, which binds the DD but not wildtype FKBP12, protects DD-tagged fusion proteins from degradation [25]. Again using the In-Fusion System, four mutants of PFKFB2 were cloned into the multiple cloning site downstream of the N-terminal DD tag and a 17 aa linker sequence: the truncation mutants PFKFB2^1−250^ (PFK2) and PFKFB2^251−518^ (FBPase2); and the kinase-dead H259A and phosphatase-dead T55V point mutants, which were generated from full length PFKFB2 [26], [31], [33], [51]. The resulting expression cassettes (DD-PFK2-IRES2-Cherry, DD-FBPase2-IRES2-Cherry, DD-PFKFB2^H259A^-IRES2-Cherry, DD-PFKFB2^T55V^-IRES2-Cherry), contained in the pEN entry vector backbone, were subsequently transferred to pAd/CMV/V5-DEST (Invitrogen) using Gateway Cloning. Purified viruses were produced in HEK293T cells by the University of Michigan Viral Vector Core. The sequence fidelity of all constructs was verified by the University of Michigan DNA Sequencing Core.

### Timelapse imaging of islet Ca^2+^ oscillations using Fura-2

Filter sets were used as previously described [50]. Islets were placed in a glass bottom chamber (54 μl volume) (Warner Instruments, Hamden, CT) on an Olympus IX-71 inverted microscope equipped with a 0.3 N.A. UPlanFL/UIS2 10X objective (Olympus). The recording solution (in mM: 137 NaCl, 5 KCl, 1.2 MgCl_2_, 2.6 CaCl_2_, 10 HEPES, 11.1 glucose; pH 7.4, 310 mOsm) was maintained at 33°C using inline and chamber heaters, and perfused at a flow rate of 0.3 ml/min, first with recording solution containing 1 µM Fura-2/AM in 0.1% DMSO (Invitrogen, Carlsbad, CA) for 12 min, followed by an equivalent washing period before monitoring [Ca^2+^]_i_ for 30 min. Fura-2 fluorescence was elicited by alternating excitation wavelengths of 340 and 380 nm at 0.2 Hz under the control of MetaFluor software (Molecular Devices, Sunnyvale, CA). Changes in intracellular Ca^2+^ were expressed as 340/380 ratio. Offline calculation of the average oscillatory periods and Ca^2+^ amplitudes were performed on the Ca^2+^ traces after linear detrending using custom macros written in Matlab (MathWorks, Natick, MA): average period was determined as the dominant peak after Fast Fourier Transform; the average amplitude was produced using a peak-finding algorithm to detect the baseline and peak of each oscillation, which were averaged for >20 min recording and measured as ΔRatio (peak over baseline).

### Co-immunoprecipitation

LR Clonase II was used to transfer PFKFB2 and GK into pcDNA6.2-V5/DEST (Gateway System, Invitrogen). For each treatment, a 6-well plate of confluent HEK293T cells (obtained from the University of Michigan Vector Core) was transiently transfected with the indicated constructs. After a 2-day expression period, cells were rinsed twice in PBS and lysed in a Dounce homogenizer in buffer containing 2% sucrose, 1 mM EDTA, and 20 mM Tris (pH 7.5). After the homogenate was centrifuged (300 *g* for 3 min) to pellet the nuclei, the supernatant was diluted 1∶1 in immunoprecipitation buffer (150 mM Tris, pH 7.4, 1 mM MgCl_2_, 0.1 mM EGTA, 2% Triton X-100). Samples were normalized for lysate volume and concentration (2–3 µg/μl) and then incubated with 10 µg of anti-V5 monoclonal antibody (Invitrogen) for 4 h at 4°C. The samples were then incubated with Protein G-Sepharose beads (Pierce, Rockford, IL) for 1 h, and washed in immunoprecipitation buffer. Finally the pellet was resuspended in SDS sample buffer and fractionated by SDS-PAGE and Western blotting. Polyclonal antibodies against PFK2 (∶:500) and GK (1∶1000) were purchased from Santa Cruz Biotechnology (Santa Cruz, CA).

### FRET Imaging

Stoichiometric FRET was performed on living Min6 cells plated on glass-bottom dishes 18 hours after transfection with Lipfectamine2000 (Invitrogen). Measurements were made on the heated stage (33°C) of an Olympus IX-71 inverted fluorescence microscope equipped with a 1.4 NA UPlanSApo 100X oil immersion objective (Olympus America, Center Valley, PA), a TILL Photonics Polychrome V xenon-lamp based monochrometer (TILL-Photonics, Grafelfing, Germany), and a Photometrics QuantEM 512sc camera (Tucson, AZ). Excitation light at 436 nm (for the donor, Cerulean) and 500 nm (for the acceptor, Citrine) was passed through a dual pass excitation filter (430/24; 500/20), polychroic mirror (89006bs, Chroma Technology Corp., Brattleboro, VT), and fluorescence emission was passed through a DualView2 beamsplitter (Photometrics) containing a dichroic mirror (T495lpxr) and emission filters (ET470/24m and ET535/30) (Chroma) to allow simultaneous two channel monitoring of donor and acceptor emission. All analyses of the acquired images were performed using Metamorph (Molecular Devices). The apparent efficiency of acceptor (Citrine) in complex (E_A_), the apparent efficiency of donor (Cerulean) in complex (E_D_) and the mole fraction of acceptor to donor (RATIO) values were determined as described in [52], with modifications of [53]. The characteristic efficiency of the linked cerulean-citrine construct was measured by fluorescence lifetime to be 0.3014 [52]. Constants (α, β, γ, ξ) were empirically determined in Min6 cells (n>10 cells) for each experiment, as described previously [52]. Apparent FRET efficiency is reported as 
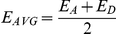
.

### The Dual Oscillator Model

The Dual Oscillator Model was developed to account for the diverse oscillatory properties of pancreatic islets [8], [38]. It consists of three interacting components representing glycolysis, mitochondrial metabolism, and plasma membrane electrical activity and Ca^2+^ handling. Slow metabolic oscillations in the model are produced by the glycolytic component, due to positive feedback onto PFK1 by its product, Fru1,6-BP. This increases PFK1 activity until the substrate level (Fru6-P) falls below the level needed to support PFK1 activity. The enzyme activity remains low until the substrate level recovers, initiating the next oscillation [41]. The glycolytic oscillations result in ATP/ADP oscillations, which in turn affect the conductance of ATP-sensitive K^+^ channels in the plasma membrane. In this way, intrinsic metabolic oscillations directly influence the electrical activity of the cell, as well as Ca^2+^ entry into the cell. The utilization of ATP to power Ca^2+^-ATPases in the plasma membrane and the endoplasmic reticulum results in turn in a lowering of cytosolic ATP. Since ATP is also an inhibitor of PFK1, this provides a pathway through which the cell's electrical activity indirectly affects the glycolytic oscillator. Thus, the two oscillators, metabolic and electrical, are bidirectionally coupled.

### The Glycolytic Component

The glycolytic component of the model was developed by Smolen [41], and all terms used in the model are described there, as well as in Bertram et al. [37]. We add to this a differential equation for the concentration of Fru2,6-BP and its stimulatory effect on PFK1. In the modified model, the concentration of Fru2,6-BP changes over time according to Eq. (5) in the Results. The PFK2 reaction term is
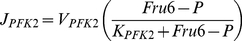
(1)and the FBPase2 reaction term is
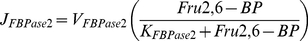
(2)where 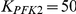 µM and 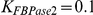 µM [54]. The maximum reaction rates (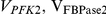) are set to 0 in the absence of PFK2/FBPase2. When PFK2/FBPase2 is present, we set 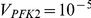 µM ms−1 and then use 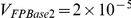 or 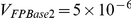 µM ms−1 to simulate the low and high ratios of PFK2/FBPase2 in Fig. 5, respectively.

Fru1,6-BP enters the PFK1 reaction term, *J_PFK1_*, as an allosteric stimulator with dissociation constant *K_Fru1,6-BP_*. Fru2,6-BP competes with Fru1,6-BP for binding to PFK1, so we replace 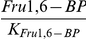 with 
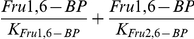
 in the expression for *J_PFK1_*. The values of the dissociation constants are from Foe et al. [14]: *K_Fru1,6-BP_* = 1.0 µM and *K_Fru2,6-BP_* = 0.15 µM.

When the glycolytic component of the DOM is run in stand-alone mode the concentration of ATP, which is inhibitory to PFK1, is a fixed parameter. In the full DOM, however, the ATP concentration varies dynamically due to mitochondrial ATP production and cytosolic ATP utilization. As a result, the stand-alone glycolytic oscillations are slower than those in the full DOM, and we increase their frequency through the parameter 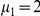 (see Eqs. (3),(4)). This parameter is not included in the DOM.

Computer codes for the stand-alone glycolytic model and the DOM are available as freeware at www.math.fsu.edu/~bertram/software/islet.

## Supporting Information

Figure S1 Full-length PFK2/FBPase2 is required for interaction with GK. (A) Co-immunoprecipitation of expressed PFK2/FBPase2 from HEK293 lysates containing coexpressed V5-tagged glucokinase (GK-V5). Anti-V5 was used for pulldown, and polyclonal antibodies against glucokinase and PFK2 were used in the Western Blots. No pulldown of PFK2/FBPase2 was observed when GK-V5 was absent (*top*). Importantly, no co-precipitation of GK was observed when FBPase2-V5 was immunoprecipitated (IP; *bottom*). Cell lysates (L) and supernatants (S) are shown for reference; FBPase2-V5 is present in the lysate, but the band is feint at the level of image scaling required for the IP band. (B) FRET measured by sensitized emission in living Min6 beta-cells transfected with PFK2/FBPase2-Cerulean and unlinked Citrine (*top*) or Citrine-GK (*bottom*). The pseudocolored E_AVG_ and RATIO images represent a spatial map of FRET efficiency and [Citrine]/[Cerulean], respectively; the color bar indicates scaling. Our optical system was calibrated before each experiment using cells expressing a linked Cerulean-Citrine construct, which was independently measured by fluorescence lifetime as having a characteristic efficiency (E_C_) of 0.30 [52], and which exhibited a measured E_AVG_ of 30.9±0.2% (n = 35). (C) Averaged E_AVG_ values for each co-transfection condition. Note that only full-length PFK2/FBPase2 interacts with GK (*left*). Furthermore, the results indicate that fluoroprotein-labeled PFK2/FBPase2 forms dimers, mediated by the interface of two opposing N-terminal PFK2 domains, while the FBPase2 domains are essentially independent (*right*). These results are expected based on previous studies of liver and testes PFK2/FBPase2 [27], [51], [55]. Comparable results were obtained when the fluoroprotein tags were moved to the opposing termini (*not shown*). The numbers in parentheses indicate the number of cells imaged. Significant differences were determined by ANOVA with a Bonferroni post-test (black bars, comparison is to PFK2/FBPase2-Cerulean plus unlinked Citrine co-transfection control), or ANOVA without a post-test (gray bars); ****, *p*<0.0001.
(TIF)

Movie S1 **3D reconstruction of a mouse pancreatic islet transduced with adenovirus.** Confocal imaging shows the infection efficiency and penetration depth of adenovirus used to deliver PFK2/FBPase2 mutants, based on expression of an IRES2-linked Cherry (red). The islet was counterstained for 1 h at 37°C with the cell-permeant dsDNA binding dye PicoGreen (green), which labeled nuclei and some mitochondria, and imaged live.
(AVI)
