# Supplementary figures and images for: Phosphofructo-2-kinase/Fructose-2,6-bisphosphatase Modulates Oscillations of Pancreatic Islet Metabolism
Source: PLoS One. 2012 Apr 20;7(4):e34036. doi: 10.1371/journal.pone.0034036 (PMC3332096; doi:10.1371/journal.pone.0034036)

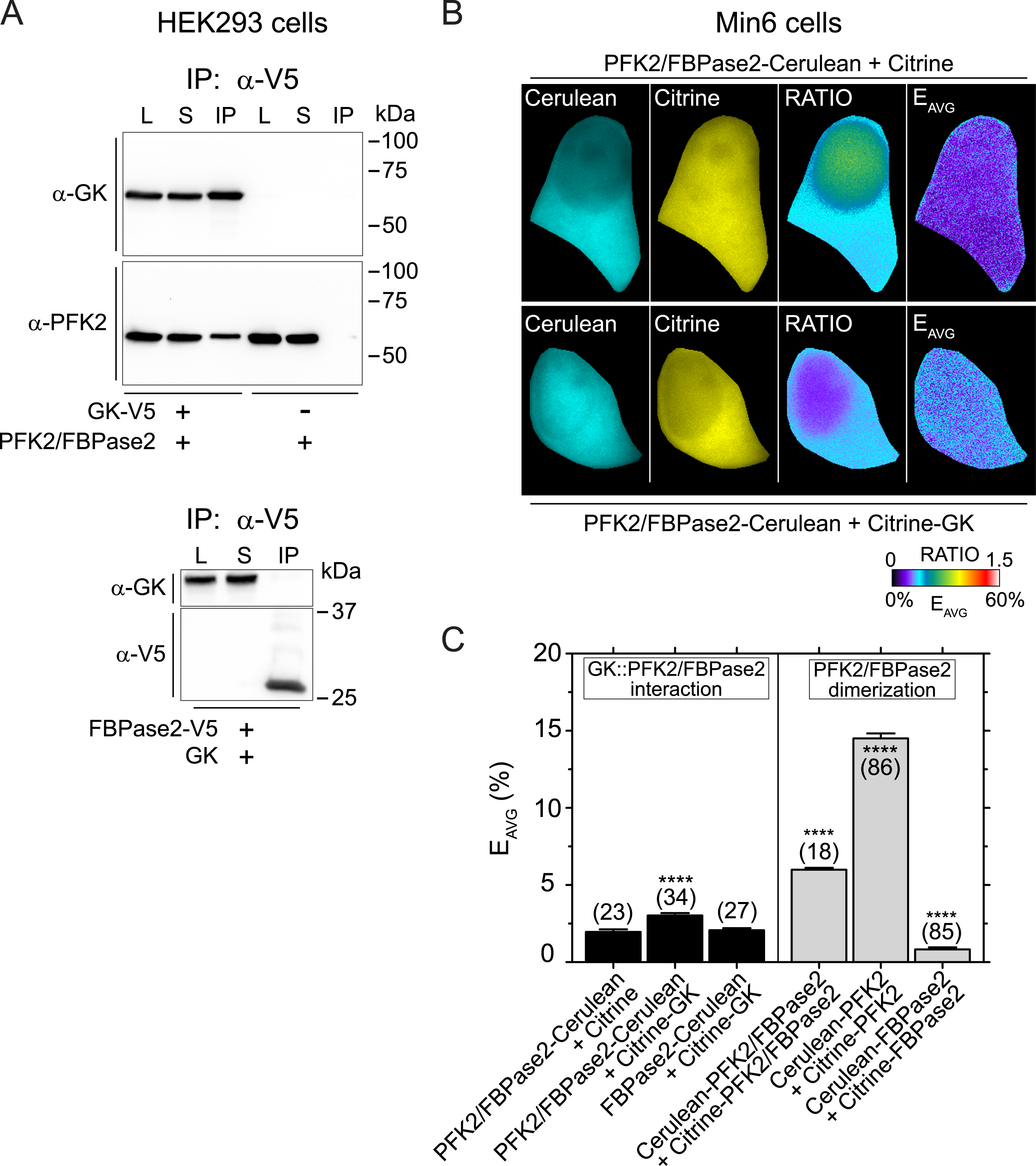

Supplement: Figure S1 — Full-length PFK2/FBPase2 is required for interaction with GK. (A) Co-immunoprecipitation of expressed PFK2/FBPase2 from HEK293 lysates containing coexpressed V5-tagged glucokinase (GK-V5). Anti-V5 was used for pulldown, and polyclonal antibodies against glucokinase and PFK2 were used in the Western Blots. No pulldown of PFK2/FBPase2 was observed when GK-V5 was absent (top). Importantly, no co-precipitation of GK was observed when FBPase2-V5 was immunoprecipitated (IP; bottom). Cell lysates (L) and supernatants (S) are shown for reference; FBPase2-V5 is present in the lysate, but the band is feint at the level of image scaling required for the IP band. (B) FRET measured by sensitized emission in living Min6 beta-cells transfected with PFK2/FBPase2-Cerulean and unlinked Citrine (top) or Citrine-GK (bottom). The pseudocolored EAVG and RATIO images represent a spatial map of FRET efficiency and [Citrine]/[Cerulean], respectively; the color bar indicates scaling. Our optical system was calibrated before each experiment using cells expressing a linked Cerulean-Citrine construct, which was independently measured by fluorescence lifetime as having a characteristic efficiency (EC) of 0.30 [52], and which exhibited a measured EAVG of 30.9±0.2% (n = 35). (C) Averaged EAVG values for each co-transfection condition. Note that only full-length PFK2/FBPase2 interacts with GK (left). Furthermore, the results indicate that fluoroprotein-labeled PFK2/FBPase2 forms dimers, mediated by the interface of two opposing N-terminal PFK2 domains, while the FBPase2 domains are essentially independent (right). These results are expected based on previous studies of liver and testes PFK2/FBPase2 [27], [51], [55]. Comparable results were obtained when the fluoroprotein tags were moved to the opposing termini (not shown). The numbers in parentheses indicate the number of cells imaged. Significant differences were determined by ANOVA with a Bonferroni post-test (black bars, comparison is to PFK2/FB [file pone.0034036.s001.tif]
